# Honokiol, an active compound of *Magnolia officinalis,* is involved in restoring normal baroreflex sensitivity in hypercholesterolemic rabbits

**DOI:** 10.1002/fsn3.1395

**Published:** 2020-01-14

**Authors:** Pei‐Yu Chou, Weng‐Cheng Chang, Fon‐Chang Liu, Shou‐Jen Lan, Ming‐Jyh Sheu, Jwo‐Sheng Chen

**Affiliations:** ^1^ Department of Nursing Hung Kuang University Taichung City Taiwan; ^2^ Sports Recreation and Health Management Continuing Studies ‐ Bachelor's Degree Completion Program Tung Hai University Taichung City Taiwan; ^3^ Department of Otolaryngology Taichung Tzu Chi Hospital Tanzi District Taichung City Taiwan; ^4^ School of Pharmacy China Medical University Taichung City Taiwan; ^5^ Department of Health and Nutrition Biotechnology Asia University Taichung City Taiwan; ^6^ Department of Sports Medicine China Medical University Taichung City Taiwan

**Keywords:** Baroreceptor reflex sensitivity, Honokiol, Hyperchlosterolemia, *Magnolia officinalis*

## Abstract

This study investigated the effects of methanol extract *Magnolia officinalis* (MEMO) on baroreceptor reflex sensitivity (BRS) in the hypercholesterolemic rabbits and the involved molecular mechanisms. Male New Zealand white rabbits were randomly divided into Control (normal diet), Cholesterol (0.5% w/w cholesterol diet), and Magnolia groups (0.5% w/w cholesterol diet plus 1% w/w MEMO). The animals were treated with the designated diet for 4 or 8 weeks. BRS in the control of heart rate was assessed by linear regression method. After 8 weeks of treatments, plasma total cholesterol (TC) was significantly elevated in the Cholesterol/Magnolia groups. The arterial blood pressure (aBP) was increased in the Cholesterol and Magnolia groups. The depression of BRS observed in the Cholesterol group was significantly ameliorated in the Magnolia group. After L‐NAME (*Nω*‐nitro‐Larginine methyl ester, 20 mg/kg, *iv*), the BRS of the Cholesterol group was significantly improved. Results from our in vitro study further indicated that honokiol, the principle component of MEMO, would protect human umbilical vein endothelial cells (HUVECs) from H_2_O_2_‐induced damages and inhibit H_2_O_2_‐induced vascular smooth muscles cells (VSMCs) proliferation, which was evident by the decreased expression of pFAK, and p‐Erk1/2. The results of the present study suggested that the improvement of BRS by MEMO in the hypercholesterolemic rabbits might be mediated by the antioxidant property of MEMO as indicated by the results from the L‐NAME and in vitro honokiol studies.

## Graphical abstract

The honokiol study implied that effects of the methanol extract Magnolia officinalis (MEMO) affect baroreceptor reflex sensitivity (BRS) in atherosclerotic rabbits.

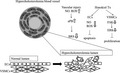

## 1 INTRODUCTION

The regulations of blood pressures and heart rates in daily activities by baroreflex were obviously depressed in patients and experimental animals with hypercholesterolemia (Minami et al., 1999; Vasquez, Peotta, & Meyrelles, 2012). Baroreflex is important in circulatory homeostasis particularly in the aBP through regulation of heart rates (HR) and vascular resistances (Fan, Reynolds, & Andresen, 1996). Mechanosensitive nerve endings of the baroreceptors are located in the carotid sinus and aortic arch, which are also the common sites of vascular lesions in atherosclerosis (Chapleau, Hajduczok, & Abboud, 1989). Baroreceptor reflex sensitivity (BRS) was found to be impaired in atherosclerotic subjects (Li, Mao, Abboud, & Chapleau, 1996). Attenuation of BRS in carotid atherosclerosis has been reported in humans (Nasr, Pavy‐Le Traon, & Larrue, 2005). Impairment of baroreflex function in hypercholesterolemia has been attributed to NO, reactive oxygen species (ROS), and other factors. It was found that reduction of vascular wall distensibility in atherosclerosis would attenuate BRS (Mattace‐Raso et al., 2007). Depression of BRS in atherosclerosis might be related to the increased vascular stiffness and lesions of baroreceptors (Li et al., 1996). Oxygen free radicals were also shown to play an important role in baroreflex dysfunction in atherosclerosis (Li et al., 1996). Previous study demonstrated that exposure of isolated carotid sinus to superoxide dismutase (SOD) and catalase increased the BRS in hypercholesterolemic rabbits (Li et al., 1996). On the other hand, when carotid sinus treated with exogenous free radicals, the BRS was also found depressed. Therefore, free radicals apparently play a significant role in baroreceptor dysfunction (Hatcher, Gu, & Cheng, 2016).

The *Magnolia officinalis* L. (*M. officinalis*) is a known antioxidant and anti‐inflammation herb which have been widely prescribed probably on a daily basis in Chinese medicine practice for centuries (Chuang et al., 2013). A 200 mg serving of magnolia bark would contain 95% of honokiol and magnolol. *M. officinalis* was used clinically for the treatment of stresses and muscle tensions (Weeks, 2009). Extracts of *M. officinalis* were also used for acute inflammation conditions (Wu et al., 2011). Anti‐inflammatory property of *M. officinalis* was found in Lipopolysaccharide (LPS)‐stimulated human gingival fibroblasts (HGF‐1) and monocytes (U‐937) (Walker et al., 2013). Free radical scavenging property of honokiol and magnolol, the active components of *M. officinalis*, was also demonstrated in α, α‐diphenyl‐β‐pricrylhydrazyl (DPPH) assay (Kim, Lee, et al., 2013). The anti‐inflammatory and antibacterial actions of honokiol and magnolol were mediated by suppression of the production of NF‐κB‐mediated cytokine in vitro (Tse et al., 2007). Magnolol was an effective vascular smooth muscle relaxant, antioxidant, and anti‐inflammatory agent (Chuang et al., 2013) and might be associated with reactive oxygen species (ROS)‐induced apoptosis and the PI3K/AKT/mTOR signaling pathway (Kim, Oh, Park, Bae, & Lee, 2013). Honokiol was shown to inhibit production of nitric oxide (NO) and LPS‐induced phosphorylation of ERK1/2, JNK1/2, and p38 (Chao et al., 2010).

Overproduction of NO and other ROS would account for the endothelial cells apoptosis and VSMCs proliferation in the development of atherosclerosis. Hyperlipidemia and elevated ROS levels might also account for the attenuation of BRS and the development of atherosclerosis. The present study was to investigate the effects of methanol extract of *M. officinalis* (MEMO) on the lipid profiles, aBP and BRS in high‐fat atherogenic diet‐induced hypercholesterolemic rabbits and the underlining molecular mechanism. Honokiol, an active compound of MEMO, was used in vitro to evaluate whether the protective effects of *M. officinalis* would be related to the protection of vascular endothelial cells and inhibition of vascular smooth muscle proliferation. Also, L‐NAME was used to determine the role of NO in the depression of BRS in the hypercholesterolemia rabbits.

## 2 METHODS AND MATERIALS

### 2.1 Chemicals

Cholesterol, bovine serum albumin, phenylephrine hydrochloride, sodium pentobarbital, heparin, DMSO, *Nω*‐nitro‐Larginine methyl ester (L‐NAME), and 3‐(4,5‐Dimethylthiazol‐2‐yl)‐2,5‐diphenyltetrazolium bromide (MTT) were purchased from Sigma‐Aldrich. Enzyme kits for serum lipid profile were purchased from Randox. Bradford protein assay was purchased from Bio‐Rad (#223‐9950). The 0.5% cholesterol supplement feed was made by dissolving 0.5 g of cholesterol in 10 ml of hot corn oil and mixing it with 100 g of ordinary rabbit chow. It was stored under 5°C and dried properly.

### 2.2 Preparation of methanol extract of *M. officinalis* (MEMO)

*M. officinalis* L. (Magnoliae Cortex; Magnoliaceae) (Ching‐Long, Tai‐chung, Taiwan) (1.5 kg) was extracted with methanol (4 × 6 L, 24 h each) at room temperature. Extracts were filtered and evaporated to dry under reduced pressure at 40°C; a dark brown residue (60 g) was obtained.

### 2.3 HPLC analysis of methanol extract of *M. officinalis*

Our previously described method was slightly modified (Pan et al., 2010). Before HPLC analysis, MEMO was filtered with a 0.2 µm Millipore filter. A total volume of 10 μL was loaded to the column. External standards of 100 μg/mL were prepared with HPLC grade‐methanol for calculation of the concentrations of examined samples. Reverse phase HPLC was performed on a HITACHI HPLC system (Tokyo, Japan) equipped with HITACHI L‐7100 pump, HITACHI L‐7400 UV detector, and HITACHI L‐7200 autosampler. LiChroCART 250–4 C18 HPLC cartridge (5 μm; Merck, Whitehouse Station, NJ, USA) was used for separations. Conditions for separation of examined samples were listed in Table 1. Based on the separation conditions of the standard, two candidate fractions were harvested (collection period: retention time ± 5 min). These fractions were further determined by electrospray ionization‐ion trap mass spectrometry (HCT ultra PTM Discovery system; Bruker Daltonics, Billerica, MA, USA) for identification of the target compounds. Capillary voltage was 4,000 V, capillary exit offset 220 V, skimmer potential 60 V, and the trap drive value was 78. Conventional data of electrospray ionization‐ion trap mass spectrometry were recorded using a scan range of 150–280 m/z. Nebulizer (nitrogen) pressure was 10 psi with dry gas flow of 5 L/min and dry temperature at 300°C.

**Table 1:** HPLC separation conditions for identifying marked components within methanol extract of *M. officinalis* (MEMO)

Compounds	Mobile phase	Wavelength (nm)	RT (min)	Contents (mg/g of MEMO)
Magnolol	ACN: H_3_PO_4_ (0.11%, pH = 2.2) = 60:40	290	10.17	110.89 ± 0.82
Honokiol			7.34	114.51 ± 0.78

Note

All samples were loading at a total volume of 10 μL into the HPLC cartridge and using a flow rate of 1.0 ml/min to perform HPLC analysis.

Abbreviations: ACN, acetonitrile; RT, retention time.

### 2.4 Animals

Male New Zealand White (NZW) rabbits (2.0–3.0 kg) were purchased from the National Laboratory Animal Center (Nankang, Taiwan). The animals were housed in the individual cage with free access to designated food and drinking water under the room temperature of 20 ± 1°C and humidity of 50%–70% for 8 weeks. Light/dark cycle was set to 12/12 hr. Blood sampling was taken prior to treatment (w = 0), at week four (w = 4) and at week eight (w = 8). The study's protocols and procedures were ethically reviewed and approved by a recognized ethical body (Institute Animal Care and Use Committee; IACUC) at China Medical University. After a one week adaptation period, animals were randomly divided into three groups. After 8 weeks of feeding with the designated diet, all rabbits were anesthetized with sodium pentobarbital (30 mg/kg, i.v.) *via* the marginal ear vein. Blood samples were also collected through the marginal ear vein.

### 2.5 Treatment protocol

The animals were divided into three groups (*n* = 8) as described below. In animals with NO synthase blockade, L‐NAME was intravenously administered 10–20 min before the phenylephrine injection.

Group 1. Control group was given 100 g/kg BW/day of normal rabbit chow (Fwusow, Taichung, Taiwan) for 4 and 8 weeks, respectively.

Group 2. Cholesterol group was given 100 g/kg BW/day added with 10% corn oil and 0.5% cholesterol for 4 and 8 weeks, respectively.

Group 3. Magnolia group was administered 100 g/kg BW/day of high‐fat atherogenic diet (10% corn oil and 0.5% cholesterol) supplemented with MEMO (1% w/w) for 4 and 8 weeks, respectively.

### 2.6 Surgical preparation

The rabbits were anesthetized with sodium pentobarbital (30 mg/kg, bolus i.v. plus continuous intravenous drops at about 6 mg kg^−1^ h^−1^). Absence of pedal reflexes indicated the adequate anesthesia. A cannula was introduced into the trachea through an incision at neck region to prevent respiratory obstruction. Importantly, rectal temperature of animals were kept at 35–36℃ with a heating plate. A polyethylene catheter (PE‐90, Becton Dickinson) filled with heparin solution (500 IU/mL, v/v) was inserted into the left femoral artery for recording of aBP. Through PE‐50, polyethylene catheters were also inserted into femoral veins of both limbs for drugs administration. The arterial catheter was connected to a pressure transducer by a three‐way stopcock.

### 2.7 aBP and HR recording

While the experiment, the catheters were maintained patent by flushing with heparinized saline (500 IU/mL) occasionally. The pressure recording system was calibrated before the experiment with a mercury manometer. The aBP was recorded continuously by a pressure transducer (model P23‐ID, Gould Instruments, Cleveland, OH, USA), amplified with a pressure processor amplifier (Gould 20–4615–52). Resting aBP was determined after a 20‐min stabilization period. Analogue signals including the systolic, diastolic, mean arterial pressures, and HR were digitized and stored on the PowerLab Data Acquisition System (4SP, AD Instruments, Castle Hill, Australia) with a computerized analysis program.

### 2.8 Measurement of BRS

BRS was measured using a linear regression method. The relationship between the peak increases in systolic blood pressures evoked by phenylephrine (0.25, 0.5, 0.75, and 1 µg/kg, *i.v.*) and the associated reflex responses in HR was analyzed by linear regression for the individual animal (Hsieh & Hong, 2008), and the slopes of the regression lines were used as the indices of BRS.

### 2.9 Measurement of plasma lipid level

Blood samples collected in EDTA‐coated glass tubes were centrifuged at 1;000 × g for 15 min at 4°C. The plasma was taken for the measurements of total cholesterol (TC) and triglyceride (TG) using the commercial analysis kits from Randox (TR212).

### 2.10 Cell lines and cultures

Human umbilical vein endothelial cells (HUVECs; BCRC number: H‐UV001) were purchased from Food Industry Research and Development Institute (Hsinchu, Taiwan). The cells were cultured on the 100‐mm gelatin‐coated dishes maintained in M199 medium (#31100‐035; Invitrogen) containing 10% FBS (#10099‐141; Invitrogen), 25 unit/ml heparin, 30 μg/mL endothelial cell growth supplements (#E0760; Sigma‐Aldrich), 2 mM L‐glutamine, 100 units/mL penicillin G, and 100 μg/mL streptomycin sulfates, 1.5 g/L sodium bicarbonate. Cells of the 3^rd^–5th passage were used. VSMCs obtained from Food Industry Research and Development Institute (Hsinchu, Taiwan) were maintained in Dulbecco's modified Eagle's medium (DMEM) supplemented with 10% (v/v) fetal bovine serum (FBS), 1 mM sodium pyruvate, 2 mM L‐glutamine 100 mg streptomycin/mL, and 100 units of penicillin. All cells were incubated at 37C and humidified 5% CO_2_. Culture media were changed every 2 days.

### 2.11 MTT cell viability assay

The protective effects of honokiol on the losses of viability induced by H_2_O_2_ in HUVECs were determined by MTT assay. In 96‐well plates, cells of 4 × 10^4^ cells/well were seeded and cultured in medium overnight. Then, the cells were exposed to various concentrations of H_2_O_2_ (0, 6.25, 12.5, 25, 50, 100, and 200 μM) in a total volume of 100 μL 10% FBS for 24 hr. A volume of 10 μL of 5 mg/ml MTT (3‐[4,5‐dimethylthiazol‐ 2‐yl]‐2,5‐diphenyl tetrazolium bromid) was added to each well. After 3 hr, the cells were washed twice with iced 1 × PBS and 100 μL of DMSO was added to each well. Absorbance values were determined at wavelength of 570 nm for each well using 650 nm as the reference. The absorbance was correlated to the percentage of vital cells compared with Control group. Cell viability for VSMCs was also evaluated by MTT assay. After cells were cultured in medium in 24‐well plates with 3 × 10^4^ cells/well overnight, they were starved for 24 hr in 0.5% FBS/DMEM. The cells were then cultured in a total volume of 500 μL culture medium containing 0.5% FBS, 200 μM H_2_O_2,_ and various concentrations (0, 1.25, 2.5, 5, and 10 μM) of honokiol for 24 hr. After 24 hr exposure to FBS, H_2_O_2,_ and honokiol, 200 μL of 0.5 mg/ml MTT was added to each well. After 4 hr of incubation, the culture medium containing the MTT solution was carefully remove by suction and 200 μL of DMSO was added to each well. Absorbance at 570 nm was determined for each well using 650 nm as the reference wavelength. The absorbance was correlated to the percentage of survival cells compared with those of the Control group (0.5% FBS).

### 2.12 Western blot analysis

A density of 5 × 10^6^ HUVECs was plated in 10‐cm dishes and cultured in media containing 6.25 μg/ml of honokiol in RPMI and 1% FBS for 2, 6, 12, 24, and 48 hr. The cells were collected, lysed in a lysis solution (6.25 ml Tris‐HCL, pH6.8. 10ml 10% Sodium dodecyl sulfate（SDS）, 8.3 ml Dl‐Dithiothreitol (DTT), incubated at 95°C for 5 min, separated in a 12% polyacrylamide gel, and then transferred onto the Polyvinylidene fluoride (PVDF) membrane. The membrane was blocked by 5% nonfat milk in PBS‐Tween20 buffer for 1 hr. It was then probed with pFAK (sc‐81493)‐ and pErk1/2 (sc‐514302)‐specific antibodies (Santa Cruz, CA, USA) overnight at 4°C. The blots were incubated with horseradish peroxidase‐linked secondary antibody for 1 hr, developed with the electrochemiluminsence (ECL) reagent (Invitrogen), and exposure to Hyperfilm (Amersham). The data were analyzed by Gel‐Logic 200 Imaging Systems, Molecular Imaging Software.

### 2.13 Statistical analyses

The data were presented as mean ± *SEM*. One‐way ANOVA followed by Student‐Newman‐Keul test was employed for statistical analysis for the differences between groups. A *p‐*value < 0.05 was considered statistically significant.

## 3 RESULTS

### 3.1 HPLC and mass spectrometry of the marked compounds in MEMO

**Figure 1:**
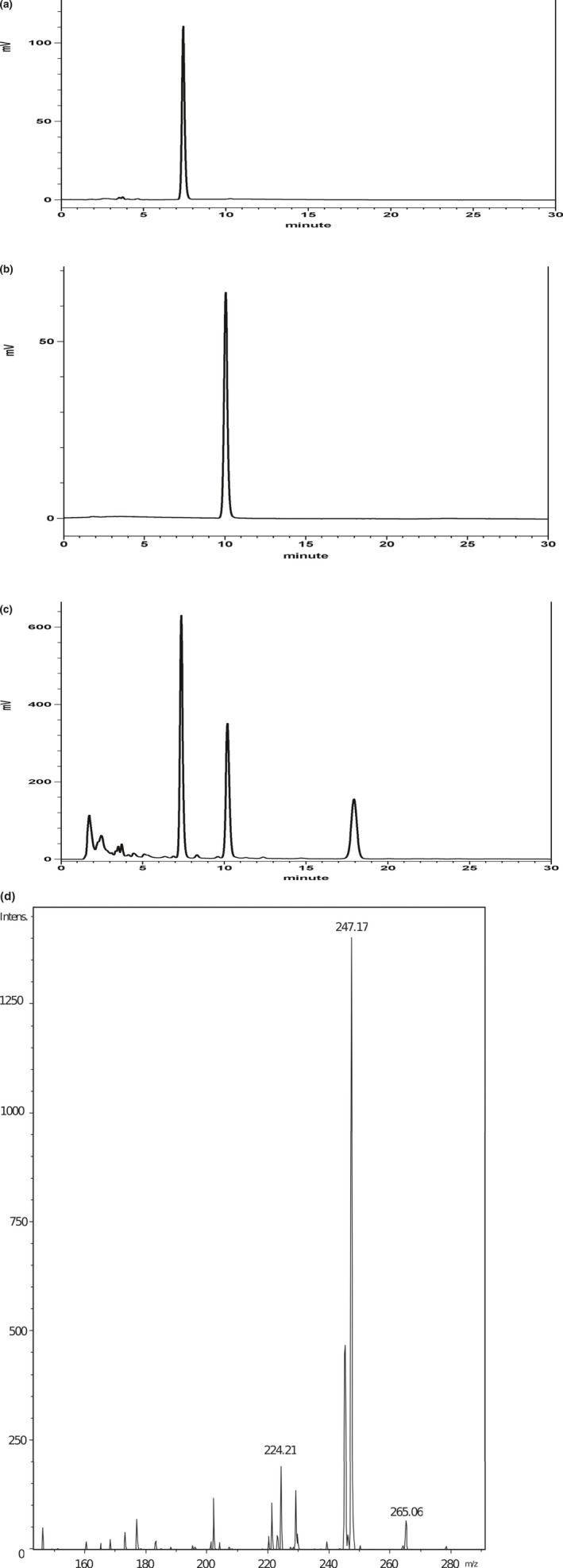
HPLC and mass spectrometry of the marked compounds in MEMO. The chromatograph indicated HPLC separation of the standard compounds (100 μg/ml) and MEMO (5 mg/ml), respectively (a, b, and c). Total volume of 10 μl was loaded into HPLC column to measure the relative content of magnolol, honokiol in MEMO according to the concentration of standard compounds. The examination conditions and monitoring wavelength of HPLC analysis were described in Table 1. (d). Three HPLC fractions (harvested period: retention time ± 5 min) were collected according to the separation conditions of standard compounds, and these fraction were further identified by ESI‐MS/MS to confirm the marked compounds (magnolol and honokiol) within MEMO

This analytic method was applied to determine honokiol and magnolol in the decoctions of MEMO. A typical chromatogram of MEMO was shown in Figure 1c. The retention times were 7.34 min for honokiol and 10.17 min for magnolol (Table 1). Identification of these compounds was confirmed by comparing the retention time with that of the standards (Figure 1a, b) and the precursor ion/product ion pair (*m/z* 265.06 → *m/z* 224.21 for honokiol; *m/z* 265.06 → *m/z* 247 0.17 for magnolol) (Figure 1d.)

### 3.2 Effects of MEMO on lipid profiles of hypercholesterolemic rabbits

As shown in Table 2, the high‐fat atherogenic diet (Cholesterol group) significantly increased serum TC levels as expected. Chow supplemented with MEMO (Magnolia group) suppressed the increases in TC caused by the high‐fat diet.

**Table 2:** Effects of on the total cholesterol of NZW rabbits fed with a high‐fat diet for 8 weeks

Group (*n* = 7)	TC (*n* = 3)	TG (*n* = 9)
Control	70 ± 5	138 ± 5
Cholesterol	781 ± 60^*^	164 ± 3
Magnolia (1% w/w)	514 ± 139^#^	191 ± 27

Note

All values are the means ± *SD*. Unit is mmole/L.

Values are presented as mean ± SE. The Cholesterol group was fed with normal rabbit chow plus 10% (w/w) corn oil and 0.5% (w/w) cholesterol. The Magnolia group was fed with the same diet as the Cholesterol group, but plus 1% w/w MEMO. ^*^ *p < *.05, compared with the Control group. ^#^ *p < *.05, compared with the Cholesterol group

### 3.3 Effect of MEMO on aBP and HR

Rabbits of the Cholesterol group showed significant rise in aBP and HR compared with control rabbits (Table 3). Compared with the Cholesterol group, the rises in aBP, but not the HR, were prevented in the Magnolia group (Table 3).

**Table 3:** Average aBP, HR, and BRS in NZW rabbits (*n* = 20) after 4 and 8 weeks of vehicle (control), cholesterol and cholesterol + MEMO administration

Groups	Control		Cholesterol		Magnolia	
Weeks	4 (*n* = 14)	8 (*n* = 21)	4 (*n* = 9)	8 (*n* = 18)	4 (*n* = 8)	8 (*n* = 12)
aBP (mmHg)	95 ± 1	96 ± 2	94 ± 2	109 ± 2^*^	101 ± 4	109 ± 2^*^
HR (bpm)	248 ± 8	255 ± 5	233 ± 10	257 ± 5	246 ± 5	274 ± 11
BRS (bpm/mmHg)	−2.22 ± 0.29	−2.26 ± 0.21	−1.71 ± 0.35	−1.28 ± 0.05^*^	−1.94 ± 0.22	−1.65 ± 0.11^*#^

Note

All values are expressed as means ± *SE*. The Control group was fed with normal rabbit chow. The Cholesterol group was fed with normal rabbit chow plus 10% (w/w) corn oil and 0.5% (w/w) cholesterol. The Magnolia group was fed with the same diet as the Cholesterol group, but plus l% (w/w) M. officinalis methanol extract (MEMO, see text for detail).^*^ *p* < .05, compared with the Control group of the same week. ^#^ *p* < .05, compared with the Cholesterol group of the same week.

Abbreviations: aBP, arterial blood pressure; HR, heart rate; and BRS, baroreceptor reflex sensitivity.

### 3.4 Effects of MEMO on the arterial baroreceptor mediated blood pressure regulatory mechanism

BRS was assessed according to the ratios of reflex bradycardic responses to the rises in arterial pressures induced by various doses of intravenous phenylephrine. BRS was significantly decreased in the Cholesterol group compared with that of the Control rabbits. Treatment with MEMO along with the cholesterol feeding significantly restored the depressed BRS in Cholesterol group (Table 3).

### 3.5 NO synthase blockade (L‐NAME) restores the BRS in hypercholesterolemic rabbits

**Figure 2:**
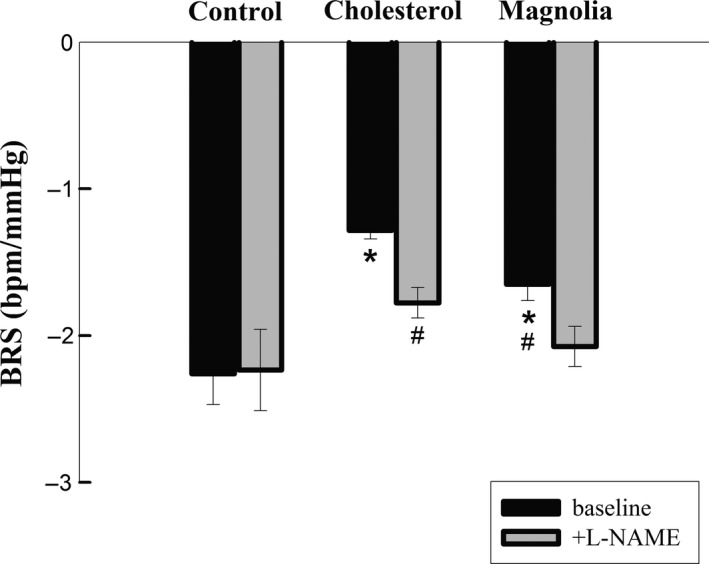
Effects of NO synthase blockade (L‐NAME) on BRS in rabbits fed with different diets. The Control group was fed with normal rabbit chow. The Cholesterol group was fed with normal rabbit chow plus 10﹪(w/w) corn oil and 0.5﹪(w/w) cholesterol. The Magnolia group was fed with the same diet as the Cholesterol group, but plus l % (w/w) MEMO (see text for detail). ^*^ *p *＜ .05, compared with the baseline values (before L‐NAME) of the Control group. ^#^ *p *＜ .05, compared with the baseline values of the Cholesterol group

To understand the role of NO on the BRS in hypercholesterolemic rabbits, L‐NAME was administered 20 min before the phenylephrine injections. BRS was attenuated in Cholesterol group (Figure 2). However, L‐NAME treatment prevented the attenuation of BRS in Cholesterol group (Figure 2). Moreover, L‐NAME further restored the BRS in Magnolia group (Figure 2).

### 3.6 Honokiol protects against H_2_O_2_‐induced cytotoxicity in HUVECs

**Figure 3:**
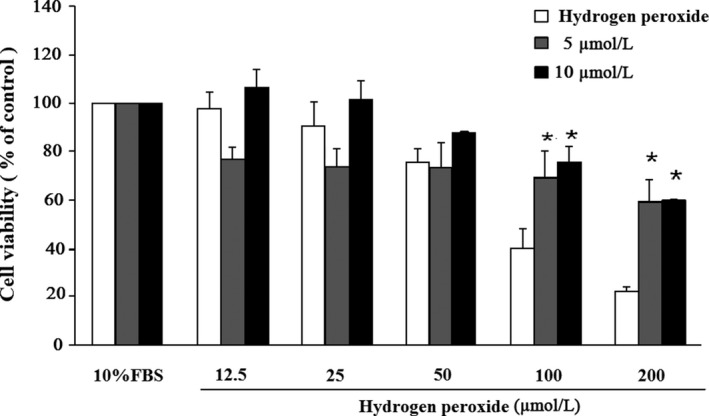
Protective effects of honokiol on HUVECs viability lost in hydrogen peroxide (HP). Cells were incubated with honokiol at concentrations of 5 and 10 μM for 24 hr, then added were the various concentrations (12.5, 25, 50, 100, and 200 μM) of HP for 12 hr. Cell viability was measured by MTT assay. The percentage of cell viability was calculated according to the values of Control group (10% FBS treated group) as 100%. Histograms of all values are expressed as the mean ± *SE* *n* = 3, ^*^ *p* < .05 as compared with Control group

HUVECs were incubated with various concentrations (5, 10, 20, 40, 60, 80, and 100 μM) of honokiol containing 10% FBS/DMEM for 24 hr. Cell viability was measured by MTT assay. It was found that honokiol at concentrations of 40–100 μM was cytotoxic to HUVECs (data not shown). To examine cytoprotective activity of honokiol, HUVECs were incubated with honokiol at concentrations of 0 ~ 10 μM containing 10% FBS/DMEM for 24 hr. HUVECs were then exposed to various concentrations (12.5, 25, 50, 100, and 200 μM) of H_2_O_2_ alone for 24 h or pretreated with two different concentrations of honokiol (5 and 10 μM) for 2 h. Cell viability was assessed using MTT assay. As shown in Figure 3, H_2_O_2_ significantly reduced the viability of HUVECs; however, honokiol at concentrations of 5 μM and 10 μM significantly increased the viability in a concentration‐dependent manner.

### 3.7 Honokiol inhibits H_2_O_2_‐induced proliferation in VSMCs

**Figure 4:**
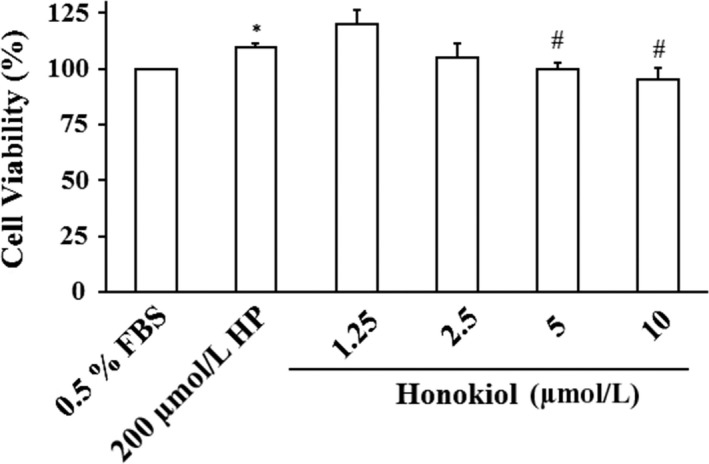
Effects of honokiol on cell viability of vascular smooth muscle cells (VSMCs). VSMCs were incubated in 200 μM HP/0.5% FBS for 12 hr pretreated with various concentrations (1.25, 2.5, 5, and 10 μM) of honokiol for 24 hr. Cell viability was measured by MTT assay. The percentage of cell viability was calculated according to the values of Control group (0.5% FBS treated group) as 100%. Histograms of all values are expressed as the mean ± *SE* *n* = 3, ^*^indicates *p* < .05 as compared with Control group. ^#^ *p* < .05 as compared with 200 μM HP

To determine the inhibitory effect of honokiol on VSMCs, the cells were incubated with various concentrations (0, 1.25, 2.5, 5, and 10 μM) of honokiol plus 200 μM H_2_O_2_ for 24 hr. Cell viability was measured by MTT assay. Our results showed that honokiol at concentrations of 5 and 10 μM significantly inhibited the VSMCs proliferation (Figure 4).

### 3.8 Expression of pFAK and pErk1/2 effected by honokiol treatment in VSMCs

**Figure 5:**
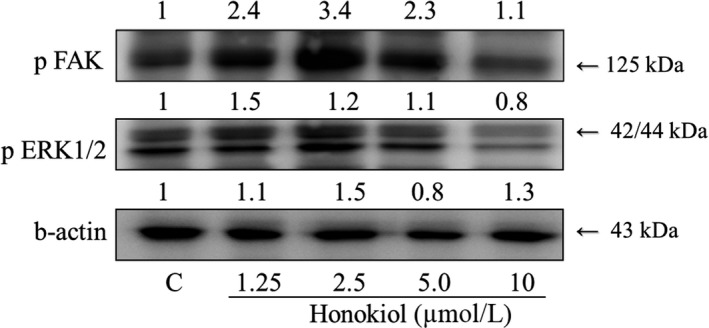
Effects of honokiol on phosphorylation FAK and ERK1/2 expression. Honokiol (1.25, 2.5, and 5.0 μM) was administered to VSMCs for 24 hr. There were concentration‐dependent effects of honokiol on the protein expression levels of phosphorylated FAK and ERK1/2. Proteins were determined by Western blotting using specific antibodies, with β‐actin detected for the control

To investigate how honokiol affected VSMCs proliferation, pFAK and pErk1/2 were measured by Western blotting assay. Our results showed that treatment with honokiol (1.25, 2.5, 5, and 10 μM) dose‐dependently reduced the phosphorylation of pFAK and pErk1/2 (Figure 5). Our data suggested that honokiol might play a significant role in regulation of VSMCs proliferation.

## 4 DISCUSSION

In the present study, we demonstrated that MEMO improved BRS (Table 3) and ameliorated the elevation of TC (Table 2) possibly by its antioxidant effects in the diet‐induced hypercholesterolemic NZW rabbits. The depressed BRS in Cholesterol group was significantly restored by L‐NAME, which indicated that the depressed BRS might be attributed to overproduction of NO, a ROS, in hypercholesterolemia (Figure 2). Honokiol, an active compound of magnolia extract, MEMO, protected the endothelial cells (Figure 3) and attenuated the proliferation of VSMCs (Figures 4 and 5). Data from in vivo study with L‐NAME, together with the results from in vitro study with honokiol, might have suggested a mechanism of action of the protection effects of MEMO. Overproduction of ROS, including NO, vascular wall inflammation and proliferation of vascular smooth muscle were the characteristic disorders of hypercholesterolemia (Elahi, Kong, & Matata, 2009; Forstermann, Xia, & Li, 2017; Orr, Hastings, Blackman, & Wamhoff, 2010). Honokiol, a known anti‐inflammatory and antioxidant agent, therefore, would have the vascular wall protection effect and further explain the vascular wall property changes‐induced BRS depression in hypercholesterolemia (Chao et al., 2010; Chuang et al., 2013). Moreover, since BRS depression in hypercholesterolemia has been attributed to the overproduction of NO (Patterson, Dick, & Struthers, 2002), antioxidant effect of MEMO (Chuang et al., 2013; Ramachandran, Wilk, Melnick, & Eliaz, 2017), therefore, might account for amelioration of the attenuated BRS.

At week 4, it was shown that aBP and HR did not increase in cholesterol diet‐feeding rabbits, which was different from previous experiments (Z. Li et al., 1996; Wilfert et al., 2000). The possible reasons might be related to the use of oil, the proportion of different cholesterol feeding time. At week 8, Cholesterol group showed a higher TC, increased aBP, and depressed BRS. When diet was supplemented with MEMO, the Magnolia group had a lower TC compared with Cholesterol group (Table 2), but the aBP was still higher than that in the Control group (Table 3). It was consistent with previous study that administration of magnolol in rabbits did not improve the hypertension status (Huang, Hong, Tsai, & Lai, 2000). However, compared with the Cholesterol group, the BRS of the Magnolia group was improved (Table 3). As previously mentioned, the protective effects of MEMO might be related to its anti‐inflammation and antioxidant properties and the possible involvement of ROS, particularly, NO.

Accumulation of oxygen‐derived free radicals would inhibit BRS during the generation of atherosclerotic arteriosclerosis (Li et al., 1996). In addition, studies have shown that chronic inflammation, the excessive accumulation of foam cells, and proliferation of vascular smooth muscle would increase vascular stiffness (Mozos et al., 2017) which would in turn change the sensitivity of baroreceptor reflex (Lee et al., 2011). NO plays an important role in the relaxation of vascular smooth muscle. Inhibition of NO would result in vasoconstriction and elevated aBP. It has been suggested that long‐term treatment with NOS inhibitor, L‐NAME, would induce hypertension, sympathetic hyperactivity, and BRS attenuation (Souza, Ballejo, Salgado, Da Silva, & Salgado, 2001). However, our data showed that acute administration with L‐NAME to hypercholesterolemic rabbits significantly restored their BRS (Figure 2). It was consistent with the findings that in the sober rabbit, acute intravenous administration of NO inhibitors also increased BRS (Liu, Murakami, & Zucker, 1996). In the present study, changes in BRS in hypercholesterolemia rabbits might be associated with NO. It was demonstrated that the antiarrhythmic effects of magnolol (an active compound in MEMO) could be inhibited by the L‐NAME. It was also suggested that the action of MEMO on BRS could be related to the NO signaling pathways (Hong, Huang, & Tsai, 1996).

Oxidative stress was thought to play an important role in the damages of endothelial cells and to be one of the etiology in atherosclerosis. ROS could induce inflammation, cell death, platelet aggregation, and VSMCs proliferation (Lee et al., 2011). These events would lead to cardiovascular lesions. It was shown that ROS‐induced injuries of endothelial cells involved in the early development of atherosclerosis (Y. Li, Liu, & Zhan, 2000). Excess ROS could cause oxidative stress and damages to plasma membranes, important cellular proteins and nucleic acid (Young & Woodside, 2001). The endothelial cells are highly sensitive to ROS, including hydrogen peroxide (H_2_O_2_). H_2_O_2_ was frequently used as an oxidative stress source in endothelial cells. It was shown that H_2_O_2_ (1–10 mM) increased intracellular Ca^2+^ and stimulated the synthesis of L‐citrulline from L‐arginine in bovine aortic endothelial cells (Shimizu, Ishii, Yamamoto, & Momose, 1997). Therefore, we also used H_2_O_2_ to investigate the effects of honokiol on the oxidative injuries in endothelial cells (HUVECs). Several studies have evaluated the antioxidant activities of honokiol. It was shown to prevent the cerebral ischemia/reperfusion injuries by modulating the enzyme systems related to ROS production and metabolism in neutrophils (Liou, Shen, Chen, Tsao, & Tsai, 2003). It also ameliorated the expression of eNOS which was depressed by oxLDL and reduced the oxLDL‐induced adhesion molecules and the adherence of THP‐1 cells to HUVECs (Ou, Chou, Lin, Yang, & Sheu, 2006). In the present study, MEMO prepared in this laboratory included two major active compounds, magnolol and honokiol (Table 1, Figure 1). Our results demonstrated that honokiol possess cytoprotective activity in H_2_O_2_‐induced injury in HUVECs (Figure 4). Honokiol at nontoxic concentrations (1.25, 2.5, 5, and 10 μM) also showed inhibition of VSMCs proliferation (Figure 5). These results suggested that honokiol might be a useful compound in the management of hyperlipidemia by both lowering TC and preventing H_2_O_2_‐induced apoptosis of HUVECs through modulating oxidative stress.

HUVECs injury would provoke migration of VSMCs. Abnormal proliferation of VSMCs played a critical role in the development of hypertension and atherosclerosis (Ross, 1999). In the present study, we determined the effects of honokiol on the oxidative injury of vascular smooth muscle cells induced by H_2_O_2_ and examined the expression of pFAK and pERK1/2 proteins in injured cells (Newby & George, 1993), which were thought to be related to the cell proliferation (Guyton, Gorospe, Kensler, & Holbrook, 1996; Zhao & Guan, 2011). Our results demonstrated that honokiol could inhibit the H_2_O_2_‐induced VSMCs proliferation.

## 5 CONCLUSION

We have successfully induced hypercholesterolemia and atherosclerosis in NZW rabbits [40]. Rabbits treated with high cholesterol diet showed significant increases in aBP and HR and significant decreases in BRS. Treatment with MEMO in addition to the cholesterol feeding prevented the rise in aBP, and HR and significant restored of BRS. After NO inhibition with L‐NAME, BRS was increased in Cholesterol and Magnolia groups. These effects might be attributed to NO. Effects of honokiol were found mediated by inhibiting ERK and FAK signaling pathways, which would subsequently prevent endothelial cells from ROS and/or NO oxidative injuries and suppressed vascular smooth muscle cells proliferation. Honokiol could be a useful compound, especially in the management of hypercholesterolemia and atherosclerosis.

## CONFLICT OF INTEREST

The authors declare that they do not have any conflict of interest.

## ETHICAL STATEMENT

This study does not involve any human testing. The animal study's protocols and procedures were ethically reviewed and approved by a recognized ethical body (Institute Animal Care and Use Committee; IACUC) at China Medical University.
